# Supplementary material for: Vertical depletion of ophiolitic mantle reflects melt focusing and interaction in sub-spreading-center asthenosphere
Source: Nat Commun. 2022 Nov 14;13:6956. doi: 10.1038/s41467-022-34781-w (PMC9663536; doi:10.1038/s41467-022-34781-w)
Supplement: Supplementary file 1 — Supplementary Information [file 41467_2022_34781_MOESM1_ESM.pdf]

Supplementary Information

Vertical depletion of ophiolitic mantle reflects melt focusing and interaction in sub-spreading-center asthenosphere

Qing Xiong [1,2 *], Hong-Kun Dai [1,2], Jian-Ping Zheng [1 *], William L. Griffin [2], Hong-Da Zheng [1], Li Wang [1], Suzanne Y. O'Reilly [2]

[1] State Key Laboratory of Geological Processes and Mineral Resources, School of Earth Sciences, China University of Geosciences, Wuhan 430074, China

[2] Australian Research Council Centre of Excellence for Core to Crust Fluid Systems (CCFS) and GEMOC, School of Natural Sciences, Macquarie University, NSW 2109, Australia

* Corresponding authors: xiongqing@cug.edu.cn (Q. Xiong); jpzheng@cug.edu.cn (J.P. Zheng).

Content summary:

(1) Supplementary Figures 1-7;

(2) Source Code 1 in the Supplementary Information;

(3) Supplementary References.

Supplementary Figures

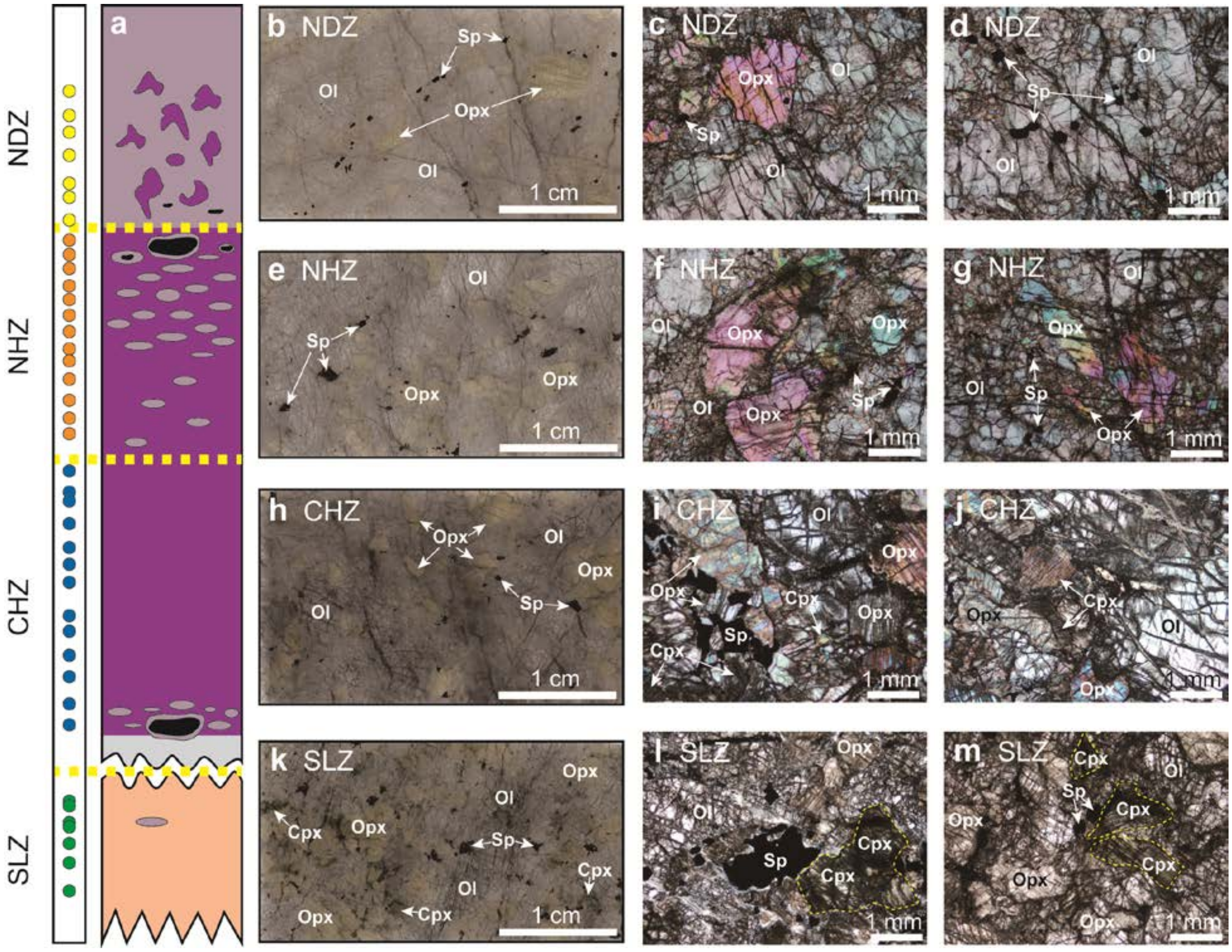

Supplementary Fig. 1. Reconstructed lithological column of the Kangjinla mantle section (**a**) and petrographic photos (**b-m**) of representative peridotites from the four zones (NDZ, NHZ, CHZ and SLZ) of the Kangjinla ophiolitic mantle (South Tibet). The legends in (**a**) are the same as those in Fig. 1. Scanned thick-section photos and microscopic images show general petrographic variations from NDZ (**b**, **c**, **d**), NHZ (**e**, **f**, **g**), CHZ (**h**, **i**, **j**) to SLZ (**k**, **l**, **m**). Microscopic images (**c**, **d**, **f**, **g**, **i**, **j**, **l** and **m**) are taken under crossed-polarizer light. Yellow dashed curves in **l** and **m** mark the porphyroblastic clinopyroxene grains in the SLZ. Abbreviations: NDZ, northern dunite zone; NHZ, northern harzburgite zone; CHZ, central

harzburgite zone; SLZ, southern lherzolite zone; Ol, olivine; Cpx, clinopyroxene; Opx, orthopyroxene; Sp, spinel.

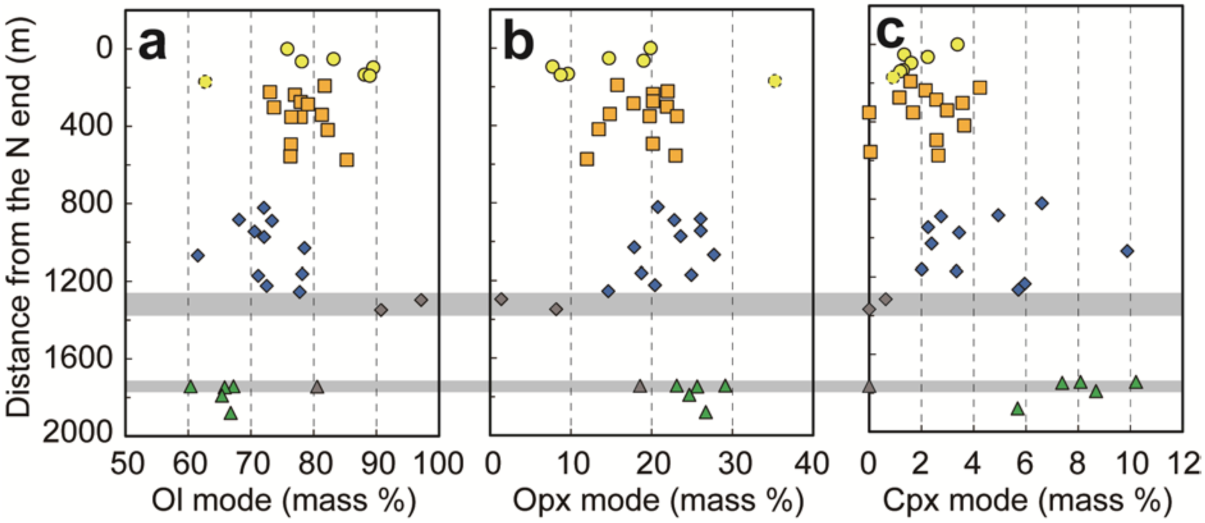

Supplementary Fig. 2. North-south variations of calculated mineral modes (**a**, olivine; **b**, orthopyroxene; **c**, clinopyroxene) of peridotites from the four zones of the Kangjinla ophiolitic mantle. Mineral modes were estimated by mass conservation calculation using whole-rock and mineral major-element compositions. The dashed yellow circles mark the sample KJL1522-04, which shows secondary addition of orthopyroxene. Grey diamond and triangle samples show strong pyroxene consumption and olivine production during melt-peridotite interaction. For the rest samples from SLZ to NDZ, the modes of olivine increase while those of pyroxenes decrease.

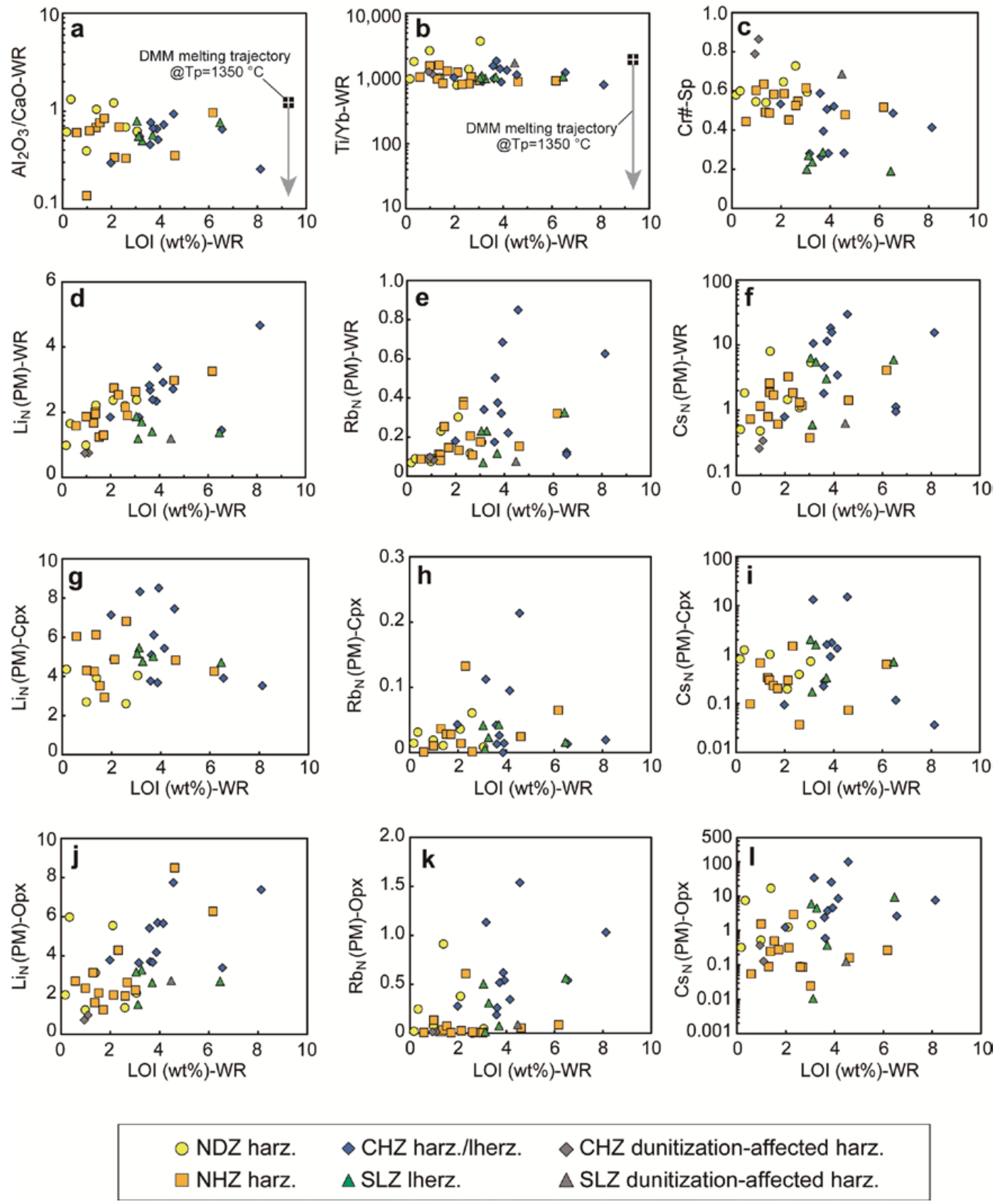

Supplementary Fig. 3. Variations of whole-rock LOI (wt%) versus whole-rock Al_2O_3/CaO (**a**), whole-rock Ti/Yb (**b**), spinel Cr# (**c**), whole-rock Li_N (normalized to primitive mantle, PM[1]; **d**), whole-rock Rb_N (**e**), whole-rock Cs_N (**f**), clinopyroxene Li_N (**g**), clinopyroxene Rb_N (**h**), clinopyroxene Cs_N (**i**), orthopyroxene Li_N (**j**), orthopyroxene Rb_N (**k**) and orthopyroxene Cs_N (**l**)

for the Kangjinla ophiolitic peridotites (South Tibet). The grey arrows in **a** and **b** represent the ranges of modeled decompressional-melting residues from a depleted-MORB-mantle (DMM) source[2] at Tp of 1350 ºC.

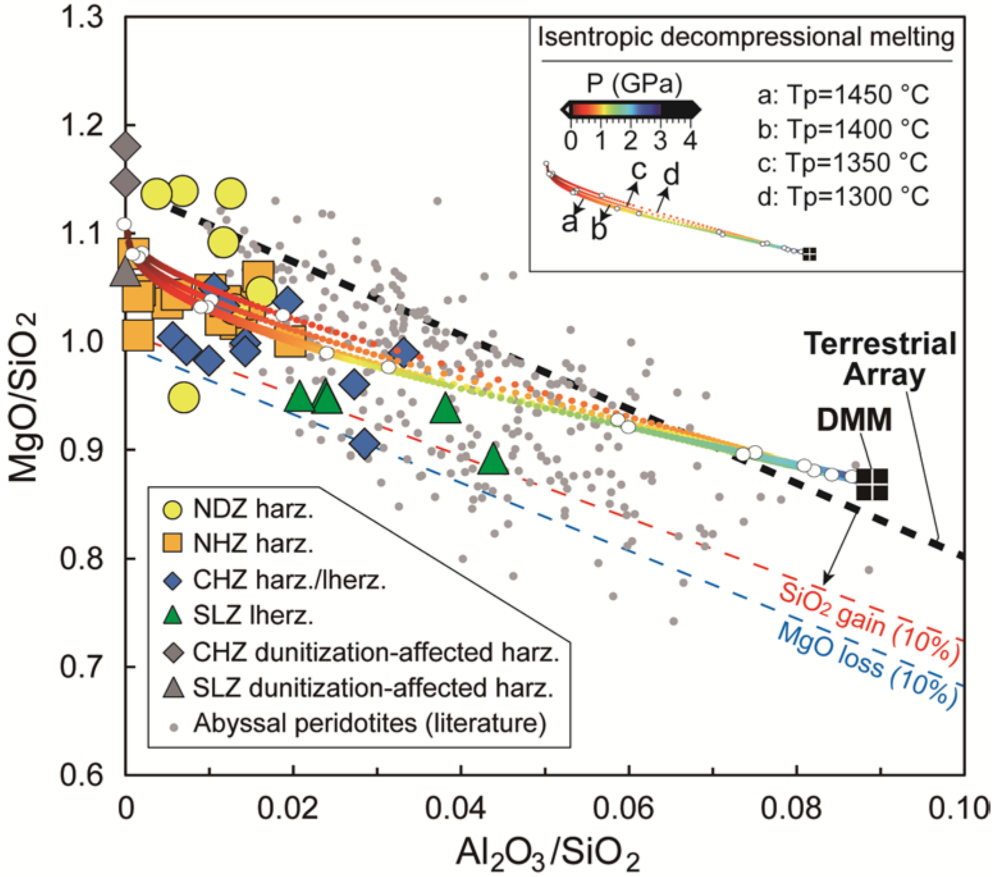

Supplementary Fig. 4. Variations of whole-rock Al_2O_3/SiO_2 versus MgO/SiO_2 for the Kangjinla ophiolitic peridotites (South Tibet). Grey diamond and triangle samples represent the Kangjinla harzburgites strongly affected by melt-peridotite interaction during dunitization. Isentropic decompressional fractional melting trends (color-coded) for residual peridotites from a depleted-MORB-mantle (DMM) source[2] were modeled using the pMELTS version[3] of alphaMELTS 1.9 program[4], with mantle potential temperatures of 1300 °C, 1350 °C, 1400 °C and 1450 °C. The color-coded pressure-decreasing gradient is of 0.1 kbar, and the white circles mark the pressure steps of 0, 0.5, 1.0, 1.5, 2.0, 2.5, 3.0, 3.5 and 4.0 GPa. The detailed melting conditions and results were listed in Supplementary Tables 10 and 11, respectively. Black dashed thick line marks the terrestrial array[5,6]. Blue and red dashed thin lines show serpentinization modification resulting in 10% MgO loss and10% SiO_2 gain, respectively, relative to the terrestrial array[7]. Small grey

circles represent global abyssal peridotites without those veined by gabbro, pyroxenite and dunite[8-10].

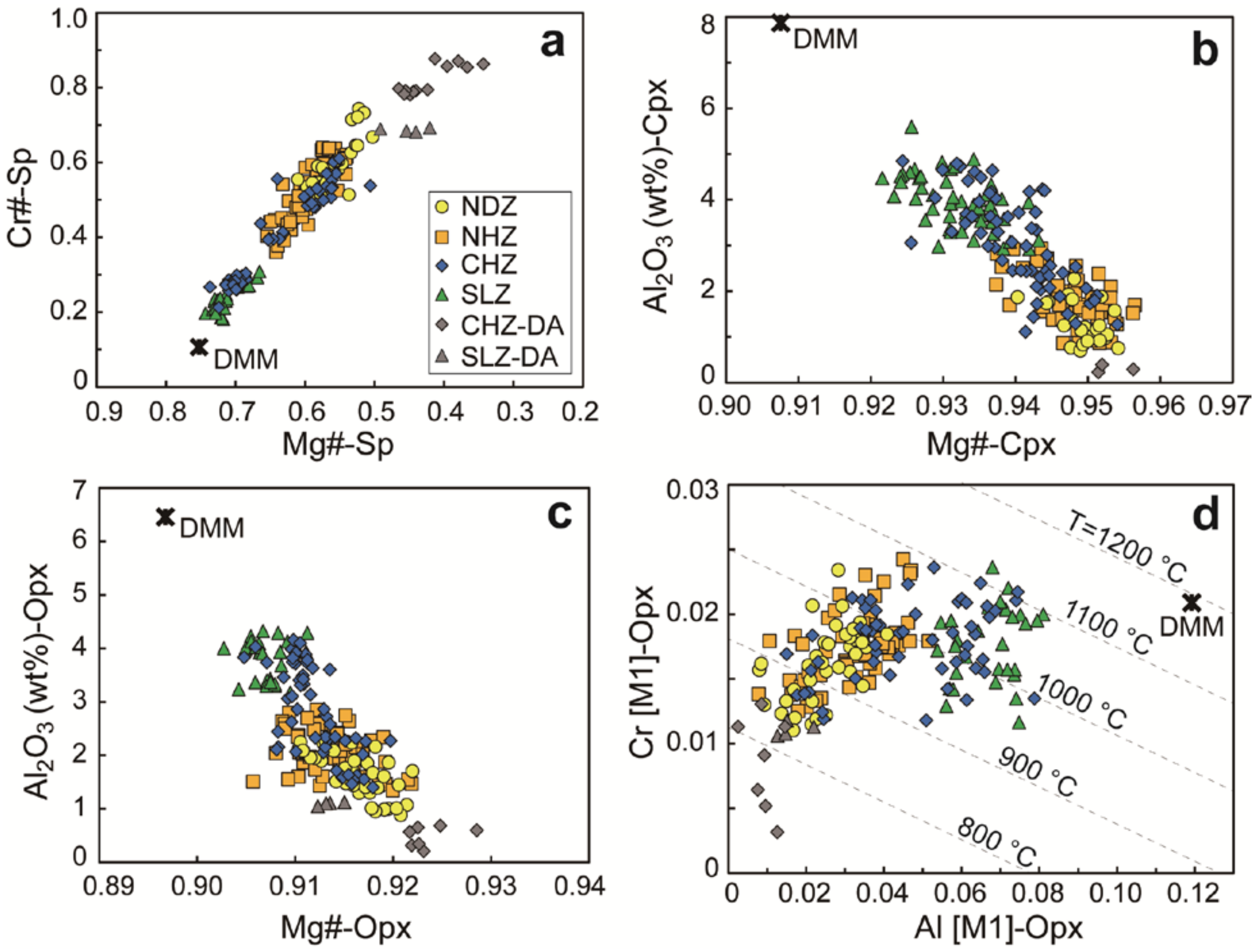

Supplementary Fig. 5. Variations of Mg# versus Cr# for spinel (**a**), Mg# versus Al_2O_3 (wt%) for clinopyroxene (**b**), Mg# versus Al_2O_3 (wt%) for orthopyroxene (**c**), and Al in [M1] versus Cr in [M1] for orthopyroxene (**d**) in the Kangjinla ophiolitic peridotites. DMM represents the minerals from the depleted MORB mantle[2]. In **d**, Al and Cr in [M1] are the Al and Cr mole fractions in the Opx [M1] site, and the black dashed lines mark the temperatures estimated using T_{WS91} thermometer of Witt-Eickschen & Seck[11]. The symbols are the same as those in Fig. 2.

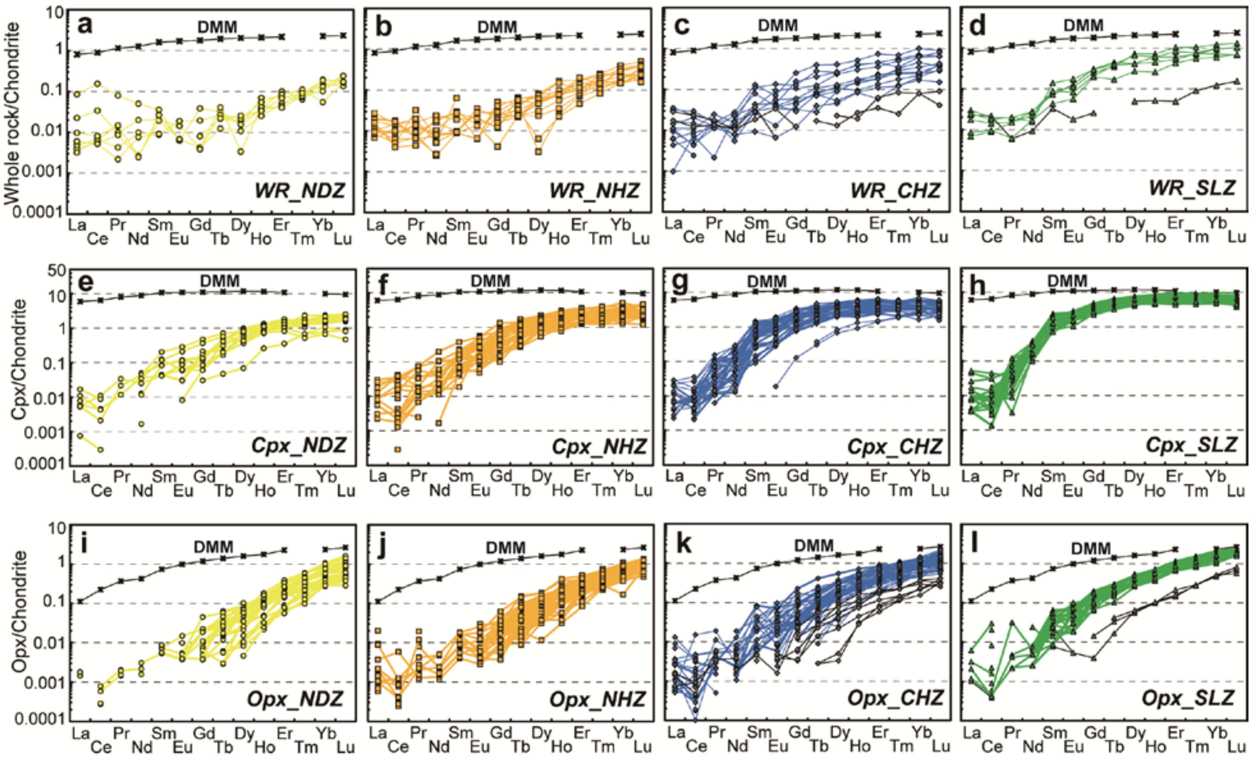

Supplementary Fig. 6. REE patterns of whole rocks (WR, **a-d**), clinopyroxenes (Cpx, **e-h**) and orthopyroxenes (Opx, **i-l**) in the Kangjinla ophiolitic peridotites, normalized to values of CI chondrites[1]. DMM in (**a-d**), (**e-h**) and (**i-l**) represents whole rock, clinopyroxene and orthopyroxene of the depleted MORB mantle[2], respectively. The symbols are the same as those in Fig. 2.

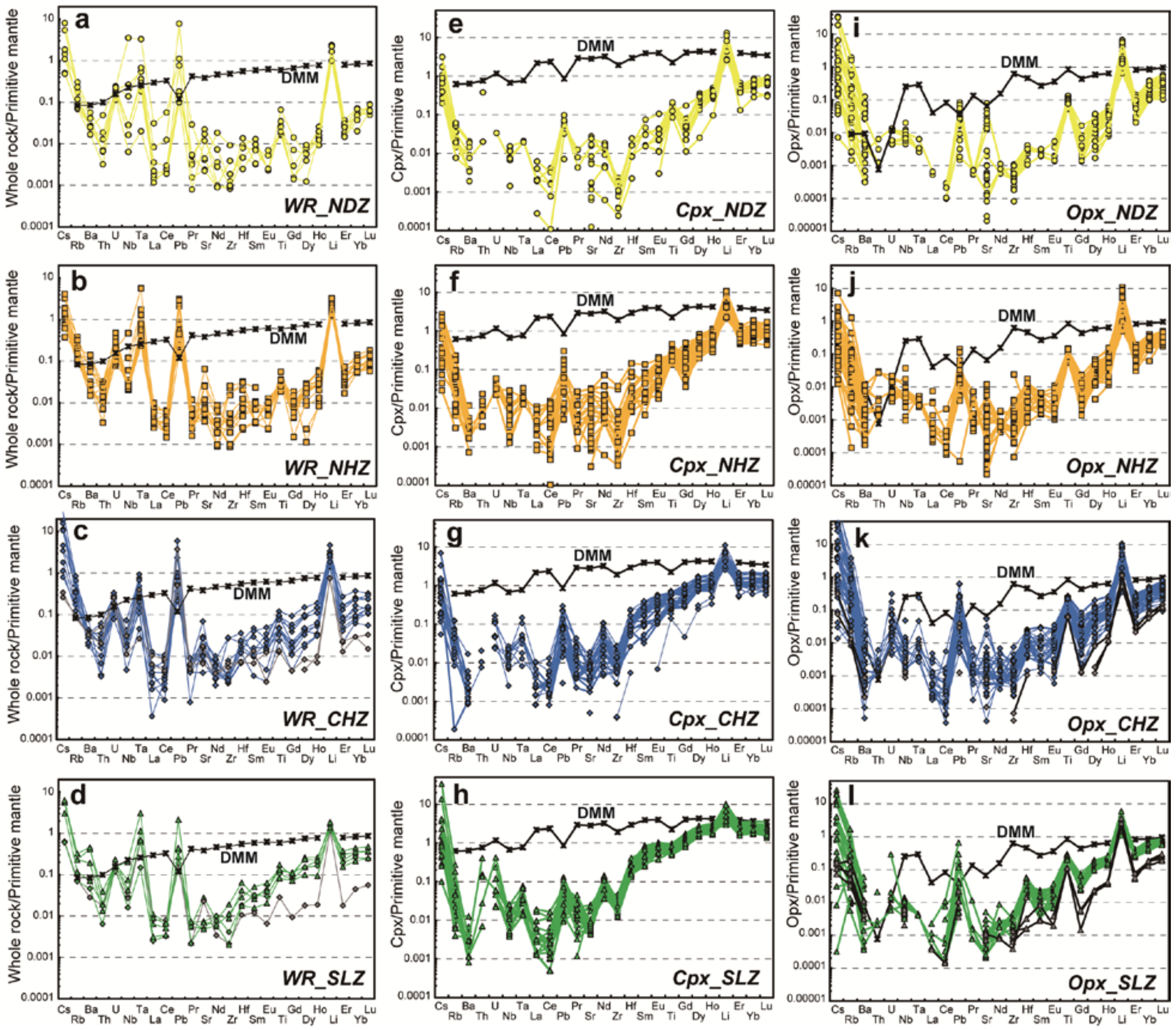

Supplementary Fig. 7. Multi-element patterns of whole rocks (**a-d**), clinopyroxenes (**e-h**) and orthopyroxenes (**i-l**) in the Kangjinla ophiolitic peridotites, normalized to values of primitive mantle[1]. DMM in (**a-d**), (**e-h**) and (**i-l**) represents whole rock, clinopyroxene and orthopyroxene of the depleted MORB mantle[2], respectively. The consistent enrichments of fluid-mobile elements across the four zones suggest pervasive metasomatism by H_2O-rich fluids after the partial melting and melt-peridotite interaction. The symbols are the same as those in Fig. 2.

Source Code 1 in the Supplementary Information

The modeling codes are included in the Source Code 1 as a compressed package.

Supplementary References

1. McDonough, W. F. & Sun, S. S. The composition of the Earth. *Chem. Geol.* **120,** 223-253 (1995).

2. Workman, R. K. & Hart, S. R. Major and trace element composition of the depleted MORB mantle (DMM). *Earth Planet. Sci. Lett.* **231,** 53-72 (2005).

3. Ghiorso, M. S., Hirschmann, M. M., Reiners, P. W. & Kress III, V. C. The pMELTS: A revision of MELTS for improved calculation of phase relations and major element partitioning related to partial melting of the mantle to 3 GPa. *Geochem. Geophys. Geosyst.* **3,** 10.1029/2001GC000217 (2002).

4. Smith, P. M. & Asimow, P. D. Adiabat_1ph: A new public front-end to the MELTS, pMELTS, and pHMELTS models. *Geochem. Geophys. Geosyst.* **6,** Q02004, doi:10.1029/2004GC000816 (2005).

5. Jagoutz, E. et al. The abundances of major, minor and trace elements in the Earth's mantle as derived from primitive ultramafic nodules. *Geochim. Cosmochim. Acta Suppl.* **10,** 2031-2051 (1979).

6. Hart, S. R. & Zindler, A. In search of bulk Earth composition. *Chem. Geol.* **57,** 247-267 (1986).

7. Malvoisin, B. Mass transfer in the oceanic lithosphere: Serpentinization is not isochemical. *Earth Planet. Sci. Lett.* **430,** 75-85 (2015).

8. Niu, Y. L. Bulk-rock major and trace element compositions of abyssal peridotites: Implications for mantle melting, melt extraction and post-melting processes beneath mid-ocean ridges. *J. Petrol.* **45,** 2423-2458 (2004).

9. Warren, J. M. Global variations in abyssal peridotite compositions. *Lithos* **248-251,** 193-219 (2016).

10. Day, J. M. D., Walker, R. J. & Warren, J. M. ^{186}Os-^{187}Os and highly siderophile element abundance systematics of the mantle revealed by abyssal peridotites and Os-rich alloys. *Geochim. Cosmochim. Acta* **200,** 232-254 (2017).

11. Witt-Eickschen, G. & Seck, H. A. Solubility of Ca and Al in orthopyroxene from spinel peridotite: an improved version of an empirical geothermometer. *Contrib. Mineral. Petrol.* **106,** 431-439 (1991).

12. McKenzie, D. & O'Nions, R. K. Partial melt distributions from inversion of Rare Earth Element concentrations. *J. Petrol.* **32,** 1021-1091 (1991).

13. Yao, L. J., Sun, C. G. & Liang, Y. A parameterized model for REE distribution beween low-Ca pyroxene and basaltic melts with applications to REE partitioning in low-Ca pyroxene along a mantle adiabat and during pyroxenite-derived melt and peridotite interaction. *Contrib. Mineral. Petrol.* **164,** 261-280 (2012).

14. Suhr, G., Seck, H. A., Shimizu, N., Gunther, D. & Jenner, G. Infiltration of refractory melts into the lowermost oceanic crust: evidence from dunite- and gabbro-hosted clinopyroxenes in the Bay of Islands Ophiolite. *Contrib. Mineral. Petrol.* **131,** 136-154 (1998).
