# Supplementary material for: Vertical depletion of ophiolitic mantle reflects melt focusing and interaction in sub-spreading-center asthenosphere
Source: Nat Commun. 2022 Nov 14;13:6956. doi: 10.1038/s41467-022-34781-w (PMC9663536; doi:10.1038/s41467-022-34781-w)
Supplement: Supplementary file 3 — Description of Additional Supplementary Files [file 41467_2022_34781_MOESM3_ESM.pdf]

Description of Additional Supplementary Files:

Supplementary Dataset 1: Sample information and petrography of the Kangjinla peridotites (South Tibet)

Supplementary Dataset 2: Whole-rock major element compositions (wt%) of the Kangjinla peridotites (South Tibet)

Supplementary Dataset 3: Whole-rock trace element compositions (ppm) of the Kangjinla peridotites (South Tibet)

Supplementary Dataset 4: Major-element compositions (wt%) of spinel in the Kangjinla peridotites (South Tibet)

Supplementary Dataset 5: Major-element compositions (wt%) of clinopyroxene in the Kangjinla peridotites (South Tibet)

Supplementary Dataset 6: Major-element compositions (wt%) of orthopyroxene in the Kangjinla peridotites (South Tibet)

Supplementary Dataset 7: Major-element compositions (wt%) of olivine in the Kangjinla peridotites (South Tibet)

Supplementary Dataset 8: Trace-element compositions (ppm) of clinopyroxene in the Kangjinla peridotites (South Tibet)

Supplementary Dataset 9: Trace-element compositions (ppm) of orthopyroxene in the Kangjinla peridotites (South Tibet)

Supplementary Dataset 10: Parameters used for pMELTS modeling of isentropic decompressional melting and melt-peridotite reaction for the Kangjinla peridotites (South Tibet)

Supplementary Dataset 11: Solid whole-rock results of pMELTS modeling of isentropic decompressional melting from the DMM source

Supplementary Dataset 12: Solid whole-rock results of pMELTS modeling of melt-peridotite reaction for the Kangjinla peridotites (South Tibet)
