# Supplementary material for: Vertical depletion of ophiolitic mantle reflects melt focusing and interaction in sub-spreading-center asthenosphere
Source: Nat Commun. 2022 Nov 14;13:6956. doi: 10.1038/s41467-022-34781-w (PMC9663536; doi:10.1038/s41467-022-34781-w)
Supplement: Supplementary file 5 — Reporting Summary [file 41467_2022_34781_MOESM5_ESM.pdf]

nature portfolio

Corresponding author(s): Qing Xiong
Last updated by author(s): Nov 7, 2022

Reporting Summary

Nature Portfolio wishes to improve the reproducibility of the work that we publish. This form provides structure for consistency and transparency in reporting. For further information on Nature Portfolio policies, see our Editorial Policies and the Editorial Policy Checklist.

Statistics

For all statistical analyses, confirm that the following items are present in the figure legend, table legend, main text, or Methods section.

n/a	Confirmed	
☐	☒	The exact sample size (*n*) for each experimental group/condition, given as a discrete number and unit of measurement
☐	☒	A statement on whether measurements were taken from distinct samples or whether the same sample was measured repeatedly
☐	☒	The statistical test(s) used AND whether they are one- or two-sided<br>*Only common tests should be described solely by name; describe more complex techniques in the Methods section.*
☐	☒	A description of all covariates tested
☐	☒	A description of any assumptions or corrections, such as tests of normality and adjustment for multiple comparisons
☐	☒	A full description of the statistical parameters including central tendency (e.g. means) or other basic estimates (e.g. regression coefficient) AND variation (e.g. standard deviation) or associated estimates of uncertainty (e.g. confidence intervals)
☒	☐	For null hypothesis testing, the test statistic (e.g. *F*, *t*, *r*) with confidence intervals, effect sizes, degrees of freedom and *P* value noted<br>*Give P values as exact values whenever suitable.*
☒	☐	For Bayesian analysis, information on the choice of priors and Markov chain Monte Carlo settings
☒	☐	For hierarchical and complex designs, identification of the appropriate level for tests and full reporting of outcomes
☒	☐	Estimates of effect sizes (e.g. Cohen's *d*, Pearson's *r*), indicating how they were calculated

Our web collection on statistics for biologists contains articles on many of the points above.

Software and code

Policy information about availability of computer code

Data collection	Modeling used the open-sourced pMELTS version of the alphaMELTS 1.9 software package. All codes have been provided in the Source Code 1.
Data analysis	Data produced by the modeling have been processed using Excel and provided in the Supplementary Dataset.

For manuscripts utilizing custom algorithms or software that are central to the research but not yet described in published literature, software must be made available to editors and reviewers. We strongly encourage code deposition in a community repository (e.g. GitHub). See the Nature Portfolio guidelines for submitting code & software for further information.

Data

Policy information about availability of data

All manuscripts must include a data availability statement. This statement should provide the following information, where applicable:
- Accession codes, unique identifiers, or web links for publicly available datasets
- A description of any restrictions on data availability
- For clinical datasets or third party data, please ensure that the statement adheres to our policy

Provide your data availability statement here.

Human research participants

Policy information about studies involving human research participants and Sex and Gender in Research.

Reporting on sex and gender	n/a.
Population characteristics	n/a.
Recruitment	n/a.
Ethics oversight	n/a.

Note that full information on the approval of the study protocol must also be provided in the manuscript.

Field-specific reporting

Please select the one below that is the best fit for your research. If you are not sure, read the appropriate sections before making your selection.

☐ Life sciences ☐ Behavioural & social sciences ☒ Ecological, evolutionary & environmental sciences

For a reference copy of the document with all sections, see nature.com/documents/nr-reporting-summary-flat.pdf

Ecological, evolutionary & environmental sciences study design

All studies must disclose on these points even when the disclosure is negative.

Study description	It belongs to Earth Sciences.
Research sample	Peridotites.
Sampling strategy	Collecting samples by geologic hammers.
Data collection	Analyzed using instruments and produced by numerical modeling.
Timing and spatial scale	Data have been collected by several analyses from 2018 to 2022.
Data exclusions	n/a.
Reproducibility	All the analyzed and modeling data can be well reproduced. Please see the Methods.
Randomization	All data have bee processed and explained in the manuscript.
Blinding	Several reference standard materials have been analyzed as unknown samples together with the samples in our study. These standards have been reported in the Supplementary Dataset.

Did the study involve field work? ☒ Yes ☐ No

Field work, collection and transport

Field conditions	-5 to 25 Celsius degrees, sometimes with rain, for a regular year.
Location	Tibet (China), with detailed coordinates in the Supplementary Dataset 1.
Access & import/export	Free to access.
Disturbance	n/a.

Reporting for specific materials, systems and methods

We require information from authors about some types of materials, experimental systems and methods used in many studies. Here, indicate whether each material, system or method listed is relevant to your study. If you are not sure if a list item applies to your research, read the appropriate section before selecting a response.

Materials & experimental systems

n/a	Involved in the study
☒	☐ Antibodies
☒	☐ Eukaryotic cell lines
☒	☐ Palaeontology and archaeology
☒	☐ Animals and other organisms
☒	☐ Clinical data
☒	☐ Dual use research of concern

Methods

n/a	Involved in the study
☒	☐ ChIP-seq
☒	☐ Flow cytometry
☒	☐ MRI-based neuroimaging
